# Performance evaluation and ranking of regional primary health care and public health Systems in Iran

**DOI:** 10.1186/s12913-021-07092-x

**Published:** 2021-10-28

**Authors:** Arash Rashidian, Nader Jahanmehr, Farshad Farzadfar, Ardeshir Khosravi, Mohammad Shariati, Ali Akbari Sari, Soheila Damiri, Reza Majdzadeh

**Affiliations:** 1 Department of Health Management and Economics, School of Public Health, Tehran University of Medical Sciences, Tehran, Iran; 2 Department of Health Economics, Management and Policy, Virtual School of Medical Education & Management, Shahid Beheshti University of Medical Sciences, Tehran, Iran; 3 Prevention of Cardiovascular Disease Research Center, Shahid Beheshti University of Medical Sciences, Tehran, Iran; 4 Non-Communicable Diseases Research Center, Tehran University of Medical Sciences, Tehran, Iran; 5 Center for Primary Health Care Management, Ministry of Health and Medical Education, Tehran, Iran; 6 Department of Community Medicine, School of Medicine, Tehran University of Medical Sciences, Tehran, Iran; 7 Department of Health Management and Economics, School of Public Health, Tehran University of Medical Sciences, Tehran, Iran; 8 Department of Health Management and Economics, School of Public Health, Tehran University of Medical Sciences, Tehran, Iran; 9 Department of Epidemiology and Biostatistics, School of Public Health, Tehran University of Medical Sciences, Tehran, Iran

**Keywords:** Efficiency, Primary health care, Public health, Program evaluation, Health care evaluation mechanisms

## Abstract

**Background:**

The present study has been undertaken with the aim to evaluate performance and ranking of various universities of medical sciences that are responsible for providing public health services and primary health care in Iran.

**Methods:**

Four models; Weighted Factor Analysis (WFA), Equal Weighting (EW), Stochastic Frontier Analysis (SFA), and Data Envelopment Analysis (DEA) have been applied for evaluating the performance of universities of medical sciences. This study was commenced based on the statistical reports of the Ministry of Health and Medical Education (MOHME), census data from the Statistical Center of Iran, indicators of Vital Statistics, results of Multiple Indicator of Demographic and Health Survey 2010, and results of the National Survey of Risk Factors of non-communicable diseases.

**Results:**

The average performance scores in WFA, EW, SFA, and DEA methods for the universities were 0.611, 0.663, 0.736 and 0.838, respectively. In all 4 models, the performance scores of universities were different (range from 0.56–1, 0.53–1, 0.73–1 and 0.83–1 in WFA, EW, SFA and DEA models, respectively). Gilan and Rafsanjan universities with the average ranking score of 4.75 and 41 had the highest and lowest rank among universities, respectively. The universities of Gilan, Ardabil and Bojnourd in all four models had the highest performance among the top 15 universities, while the universities of Rafsanjan, Ahvaz, Kerman and Jiroft showed poor performance in all models.

**Conclusions:**

The average performance scores have varied based on different measurement methods, so judging the performance of universities based solely on the results of a model can be misleading. In all models, the performance of universities has been different, which indicates the need for planning to balance the performance improvement of universities based on learning from the experiences of well-performing universities.

## Background

The complexity of health organizations, increase of health expenditure, specialization trends, prioritization of customers, and importance of effectiveness and efficiency of services encourage health organizations to apply the performance evaluation [1], so, the demand for information for performance improvement, accountability, and stakeholder decision-making is increasing [2]. Although formal discussions for the collection and publication of performance information have been developed over the past 100 years, their wide application encounters professional, practical and political barriers in health care [3].

Performance evaluation is often used with an ambiguous concept of it in the health care sector [4] and despite the existence of different methods and frameworks for evaluating the performance of health care organizations, there is no consensus in the case of a proper approach concerning performance evaluation in this sector. There is an endless interest in the design and application of a combination of methods and frameworks for measuring the performance of these organizations [5]. With regard to the obvious relationship between the definition of technical efficiency and the definition of health systems performance, efficiency has been expressed as the most important and prevalent mechanics and key for measurement and evaluation of the performance [1, 6, 7]. The first effort made for evaluating the performance of health systems by applying the concept of efficiency was made by WHO in 2000; having evaluated the efficiency of health systems in 191 countries of the world [8]. The studies made by Hollingsworth [9], Kumbhakar [10], Cheng and Zervopoulos [11], and Grigoli and Kapsoli [12] are of additional efforts made at global level. The comparisons have been performed at restricted scales such as the level of Organization for Economic Co-operation and Development and the European countries [13–18]. The effort for the analysis of performance of health systems has declined in different regions inside some countries, especially developing countries, where the differences in the health achievements of different regions are totally obvious [19]. The timely management of performance and monitoring and the evaluation of the programs by guaranteeing the continuous improvement of programs is considered to be an important factor in the reduction of regional inequalities in health outcomes [20]. Samples of such studies are observed in Spain [21], Germany [22, 23], India [19, 24, 25], Ghana [26], Brazil [27], Kenya [28], South Africa [29], and Mozambique [30].

During recent years, the subject of performance evaluation has been considerably reflected upon in the great policies of the country, and has been emphasized on in the Ministry of Health and Medical Edjucation (MOHME) [31–34]. The execution of these rules in practice and creation, of information management systems, monitoring systems, and performance evaluation with the aim of using its innumerable advantages in the health system need special attention and is the key role of the MOHME in this area [35].

In Iran, during the recent two decades, many national researches have been accomplished about health and population [36–39]. Numerous resources are used in such studies and a large volume of data has been gathered, but there is no considerable use of them [40], whereas these data can be used in the measurement and management of performance of the health systems.

In the health system structure of Iran at the second and third care levels, the performance evaluation systems, having been developed from 1962 with a few standards, were considered completed at the time [41]. Nevertheless, the design and execution of performance evaluation systems is lacking in the structure of primary health care in the country today; while the nonexistence of some structures for managing the performance is one of the radical reasons for low efficiency and weak quality of primary health care [42].

The Universities of Medical Sciences in Iran, as the largest organizational units of the health system, play an important role as administrators of community health in the provision and expansion of health services and sustainable development of the country [43]. Monitoring and measuring the performance of health deputies in these universities are of special importance as they oversee the widest area of the health system concerning expansion, activity volume, and servicing scope in the country, as well as governing over the needed health resources. The decision-makers, at all levels, need the qualifications to recognize the differences in performance of the health system, identify those factors that are effective, and adjust policies, which lead to better results in different environments. In this regard, the performance of components in the system, such as different regions inside the country, also needs to be evaluated. The meaningful and comparable information in the case of performance of the health system, and the major agents that clarify the difference in the performance of different systems, can lead to scientific policymaking in the health system at both national and regional levels [6].

In previous studies, various approaches like ratio analysis, least-squares regression, total factor productivity, stochastic frontier analysis and data envelopment analysis have been used separately to evaluate performance with an efficiency-based approach (1–4). So, due to the limitations of each of these models, factor analysis with a combination of existing methods has been used together to present the results and a big picture of performance with different hypotheses here.

The present study has been undertaken with the aim of assessing performance evaluation and ranking the universities of medical sciences that are responsible for providing first level services, such as public and primary health care, to the general population [44].

## Methods

### Data and conceptual framework

**Fig. 1:**
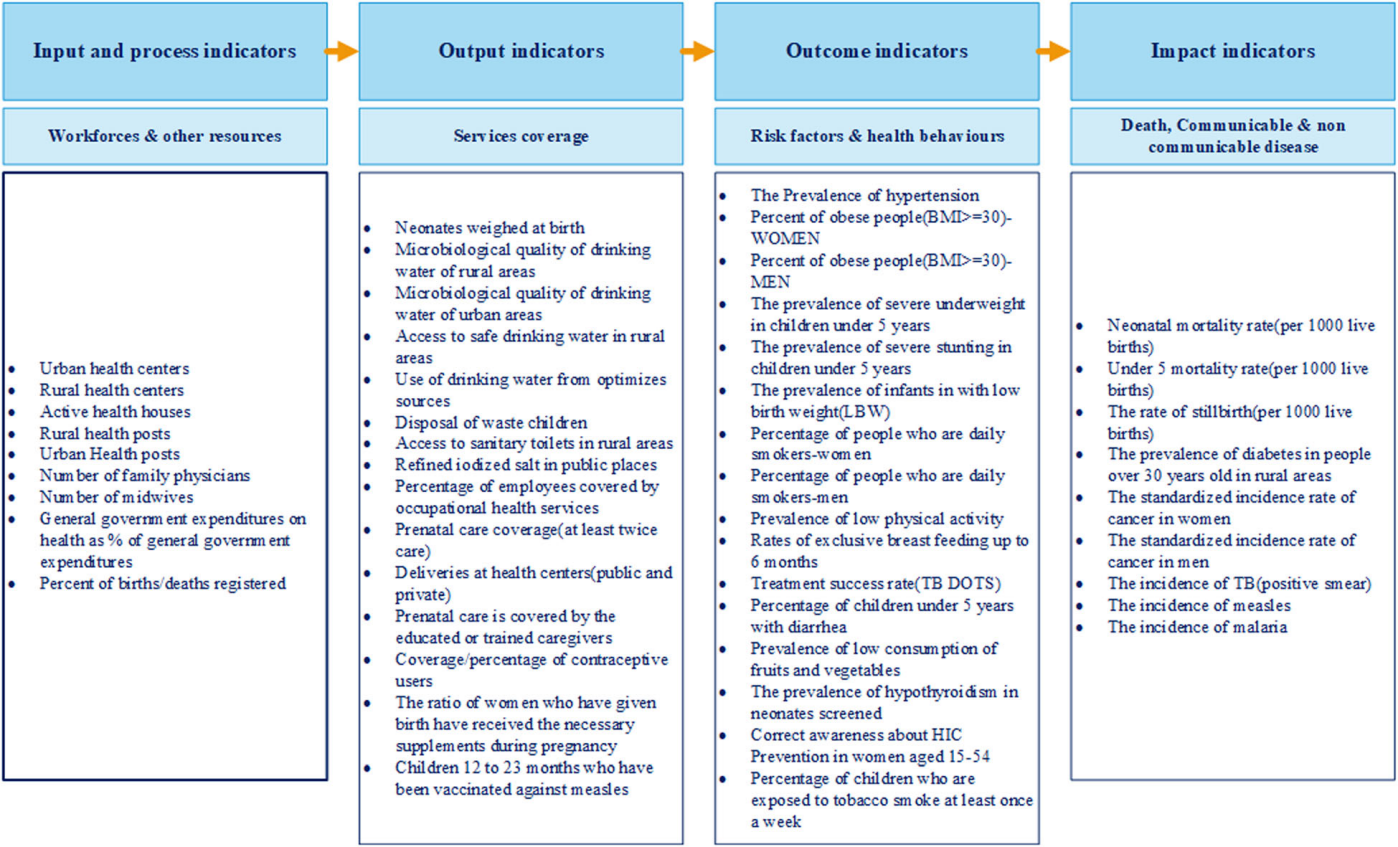
Conceptual framework of performance evaluation of primary health care and public health systems in Iran

In this study, a conceptual framework was designed to match the achieved evaluation to the structure and specific programs of the health deputies of the universities of medical sciences in Iran; exact details of methodology of its extraction having been presented in a study by Jahanmehr and his colleagues [45]. To facilitate the reporting and interpretation of the results, the term “University” is used to refer to the primary health care and public health systems in which services are organized in the form of activities developed by the health deputies of the universities of medical sciences. The health deputy of each university is responsible for providing primary health care and public health services to the population living in a region in their realm. The evaluation and ranking of the performance of public health deputies of 45 universities of medical sciences in the country was conducted based on 2010 data. This conceptual framework includes two major sections; namely health determinants and results-chain. The results-chain forms several levels including the input indicators based on human resources and health centers, the output indicators based on indicators of health services coverage, the outcome indicators based on health behaviors and risk factors, and the impact indicators based on death and communicable and non-communicable diseases (Fig. 1).

The data of the present research were collected from statistical reports of MOHME, census data of Statistical Center of Iran (SCI), indicators of Vital Statistics, results of the Multiple Indicator of Demographic and Health Survey [46], and results of the National Survey of Risk Factors of non-communicable diseases [47]. There is a university of medical sciences in every province, but in some cases, this is not the case. For cases where the data of one indicator did not exist by university, the provincial data of that indicator were generalized to the universities of that province.

### Models of performance evaluation

In the present study, four models including Weighted Factor Analysis (WFA), Equal Weighting (EW), Stochastic Frontier Analysis (SFA), and Data Envelopment Analysis (DEA) have been used simultaneously and in parallel form for measuring and ranking the performance of the health deputies.

The complexity of health systems complicates their performance summarization on a unique scale; therefore, the use of composite indexes has been popular. These indexes combine different functional indicators inside a unique index and, because they provide a wider and more comprehensive image of the performance, are used for ranking or comparing different organizations and systems [3]. In this study, a composite index has been applied for each of the models. This composite index has been obtained based on weighting of different levels of the results-chain (level of process and output, outcome and impact indicators).

The WFA and EW indicate the status of performance and total ranking of health deputies without regarding the production resources. In WFA, for obtaining the final composite index, the indicators weight has been considered different and were regarded equal in the EW model. Furthermore, this separation and weighting has been used separately in different domains of the performance in the studies performed in other countries for extracting the composite index [48, 49]. Stochastic frontier analysis and data envelopment analysis are among the most reliable empirical approaches to measuring efficiency, which were used in this study in the form of models 3 (SFA) and 4 (DEA), respectively, with the aim of measuring the performance. These two methods are different in their approaches to the creation of place and the form of production frontier, and estimation of the status of each system in comparison to that frontier. In some empirical studies, one or both of these methods are used for estimating the efficiency [50].

In models 3 and 4 in this study, factor analysis was used to extract the composite indicators required for stochastic frontier analysis and data envelopment analysis.

#### Model 1. Weighted factor analysis (WFA)

The number of primary indicators inserted in the WFA model was 41 cases (16 output indicators, 16 outcome indicators, and 9 impact indicators). In this model, a proper weight was first allocated to all the indicators by the use of factor analysis and then, three indexes of performance were obtained separately sorted by different levels of the results-chain. In the next step, the composite index of performance of health deputies of the universities was extracted by combining three performance indexes with different weights obtained from further factor analyses.

The main purpose of this method is correlating variables of regular simplification of many numbers into a few numbers of factors. From among different methods of factor analysis, the Principal Component Analysis (PCA) was selected for extracting the factors. The sampling adequacy was studied to achieve the reliable outcomes by the Kaiser-Meyer-Olkin (KMO) test and the suitability of data for reduction and execution of factor analysis was surveyed by Bartlett’s Test of Sphericity. The selection of numbers of factors was accomplished based on the criteria of Eigen Value > 1, on the explanation of more than 10% variance of all the data by each selected factors, and the explanation of more than 60% variance in all data, and was based on the outcomes of the Scree Plot test. After the calculation of the score for performance of each university by using factor analysis, to represent the amount of dispersion of each university from the country mean, based on the following equation the standardized score of each university was considered as the basis of the final ranking:

$$ \mathrm{Standardized}\ \mathrm{score}\ \mathrm{of}\ \mathrm{each}\ \mathrm{university}=\frac{\mathbf{score}\ \mathbf{of}\ \mathbf{each}\ \mathbf{university}-\mathbf{country}\ \mathbf{mean}}{\mathrm{standard}\ \mathrm{deviation}.} $$

#### Model 2. Equal weighting (EW)

The evaluation logic, analysis levels, and the indicators studied in this model are similar to WFA only with the difference that the basis for performance evaluation in EW is the allocation of equal weight to the indicators existing inside every level and was from the accomplished analyses.

Among the four evaluation models, the reason for application of EW, may be questioned due to its simplicity, because the other three models are popular among specific groups due to their relative complexity of methodology, and each one represents many researches in different scopes especially health and treatment [51–56]. The aforementioned question should be answered in this way, that many studies have already been completed by EW, and in different studies this has been defended due to its simplicity with equal emphasis on the indicators [57, 58]. Some researchers believe that although there is an ideal tendency toward the application of different weight values to the indicators based on their degree of importance, there is no reliable basis and criterion in this regard [59]. According to the mentioned background [58], this model was also applied.

For calculating the amount of technical efficiency of health deputies in models 3 and 4, it is assumed that the output or outcome of each of principal components of the indicators in the results-chain (outputs, outcomes, and final impacts), is a subject of input indicators. With regard to the surplus of number of output indicators in each component of the results-chain, factor analysis was used for the reduction and combination of indicators and the extraction of indicators needed for each evaluation level.

#### Model 3. Stochastic frontier analysis (SFA)

Fifty-three primary indicators were inserted in this model. First, the number of indicators at every level was reduced to one indicator by factor analysis, and then, performance indexes (degree of technical efficiency) were obtained separately by Stochastic Frontier Analysis and sorted by different levels of the results-chain. Selected factor was based on the criteria of Eigen Value > 1 and the explanation of more than 50% variance in all data. After combining the three performance indexes at different levels (output and process, outcome and impact) of the results-chain, the composite index was extracted of performance of those health deputies of the universities. We used Stochastic Frontier Analysis as a method for obtaining the frontier functions needed in the measurement of technical efficiency. The Maximum Likelihood Estimation (MLE) was applied for calculating non-efficiency where efficient estimations are presented for the coefficients of β parameter.

The fundamental structure of model of Stochastic Frontier production function in our study is as following:

$$ \mathrm{Y}=\upbeta\ \mathrm{X}+\mathrm{V}\hbox{-} \mathrm{U} $$

In such a way that:

$$ {\displaystyle \begin{array}{l}V\sim N\left(0,{\delta}_v^2\right)\\ {}U=\parallel U\parallel, U\sim N\left(0,{\delta}_u^2\right)\end{array}} $$

β^’^ X = definite component V = error term (Stochastic)

U = impacts of non- efficiency Y = results of health services in health deputy

X = vector of inputs β = vector of parameters

V indicates the usual error and explains the factors which are out of the control of the health deputy (such as social-economic indicators); U is indicative of non-efficiency that resulted from the issues such as weakness in the skills or slackness of management and employees or informational restrictions. In this study, we used the STATA 12 software for SFA analysis and estimation.

#### Model 4. Data envelopment analysis (DEA)

Forty-seven primary indicators were inserted in this model. First, factor analysis was performed, the number of indicators at every level was reduced 3 indexes, and then, the performance index (degree of technical efficiency) was obtained separately by applying the data envelopment analysis sorted by different levels of indicators in the results-chain. The composite index of performance of health deputies of the universities was extracted after combining these three performance indexes at different levels (output and process, outcome and impact) of the results-chain.

The data envelopment analysis is a technique of linear planning that calculates the technical efficiency separately sorted by every university using a set of optimizations and under the assumptions of minimization of production factors with a constant return to scale. In this study, the input-based DEA analysis method was used based on following equation:

$$ {\mathit{\operatorname{Min}}}_{\lambda, OS, IS}-\left(M{1}^{\acute{\mkern6mu}} OS+K{1}^{\acute{\mkern6mu}} IS\right) $$

$$ st:-{y}_i+ Y\lambda - OS=0 $$

$$ \theta {x}_i- X\lambda - IS=0 $$

$$ N{1}^{\acute{\mkern6mu}}\times \lambda \le 0,\lambda \ge 0, OS\ge 0, IS\ge 0 $$

In the stated equation, OS is the vector of output slacks; dimensions M × 1, and *IS* is the vector of input slacks; dimensions K × 1, and M_1_ and K_1_ are the unit vectors; dimensions M × 1 and K × 1. Furthermore, in the linear planning, x_i_ and y_i,_ respectively, are the input and output vectors in i^th^ and y^th^ Universities. X is the input matrix; dimensions 7 × 43, and Y is the output matrix; dimensions 3 × 43. In addition, *θ* is the criterion of technical efficiency of the input in this model, which selects the values of 0 to 1. If *θ* be equal to 1, This means that the university that is on the production function is efficient. we used the DEAP 2 software for DEA analysis.

## Results

Table 1 presents the score of performance of the health deputies of universities of medical sciences in Iran based on the results of different models. At the composite index level, the average score of performance of the universities in WFA, EW, SFA, and DEA is 0.611, 0.663, 0.736, and 0.838, respectively. In the WFA and EW models, Ilam University and Rafsanjan University showed the best and worst performance, respectively. In the SFA model, the University of Isfahan and Markazi with the score 1, and Zahedan University with the score 0.346 had the best and worst performance, respectively. In DFA model, the universities had the high level of performance in comparison to the other models, and Universities of Ardebil, Bojnourd, Bushehr, Dezful, Gilan, Gonabad and Kordestan have obtained the score 1. The lowest score in this model was related to Kashan University with a score of 0.371.

**Table 1:** Comparison of rank and composite index of performance of universities in 4 evaluation model

Rank	WFA		EW		SFA		DEA	
	University	Score	University	Score	University	Score	University	Score
**1**	Ilam	1.000	Ilam	1.000	Isfahan	1.000	Ardabil	1.000
**2**	Bushehr	0.965	Bushehr	0.950	Markazi	1.000	Bojnourd	1.000
**3**	Birjand	0.863	Golestan	0.935	ChaharM & Bakhtiari	0.971	Bushehr	1.000
**4**	Gilan	0.840	Qom	0.834	Gilan	0.966	Dezful	1.000
**5**	Golestan	0.819	Zanjan	0.804	Sabzevar	0.949	Gilan	1.000
**6**	Shahrud	0.787	Gilan	0.802	Shahid-Beheshti	0.949	Gonabad	1.000
**7**	Ardabil	0.760	Birjand	0.797	Tehran	0.939	Kurdistan	1.000
**8**	ChaharM & Bakhtiari	0.756	Bojnourd	0.786	Kermanshah	0.923	Lorestan	0.990
**9**	Bojnourd	0.749	Mazandaran	0.770	Ardabil	0.918	Neyshabur	0.986
**10**	Mazandaran	0.733	Ardabil	0.766	Qom	0.908	Qom	0.983
**11**	Azerbaijan-West	0.725	ChaharM & Bakhtiari	0.742	Kashan	0.907	Shahid-Beheshti	0.982
**12**	Fasa	0.710	Shahrud	0.725	Bojnourd	0.899	Tehran	0.976
**13**	Hamadan	0.697	Kermanshah	0.718	Azerbaijan-Eeast	0.889	Torbat-Heidariye	0.970
**14**	Lorestan	0.666	Sabzevar	0.706	Hamadan	0.887	Kohgiluyeh & BoyerA	0.966
**15**	Kermanshah	0.641	Qazvin	0.689	Yazd	0.873	Golestan	0.955
**16**	Gonabad	0.637	Isfahan	0.676	Qazvin	0.868	Hamadan	0.954
**17**	Sabzevar	0.626	Babol	0.653	Ilam	0.867	Zanjan	0.941
**18**	Shiraz	0.621	Gonabad	0.651	Golestan	0.867	Zahedan	0.884
**19**	Isfahan	0.606	Azerbaijan-West	0.645	Azerbaijan-West	0.843	Hormozgan	0.879
**20**	Mashhad	0.605	Fasa	0.624	Shiraz	0.833	Kermanshah	0.853
**21**	Jahrom	0.600	Mashhad	0.601	Kurdistan	0.804	Mashhad	0.841
**22**	Torbat-Heidariye	0.598	Hamadan	0.599	Shahrud	0.769	Zabol	0.826
**23**	Babol	0.593	Shiraz	0.585	Mazandaran	0.762	Mazandaran	0.810
**24**	Zanjan	0.593	Torbat-Heidariye	0.576	Lorestan	0.757	Ilam	0.798
**25**	Kurdistan	0.585	Kohgiluyeh & BoyerA	0.575	Mashhad	0.757	Azerbaijan-West	0.796
**26**	Azerbaijan-Eeast	0.582	Hormozgan	0.575	Zanjan	0.755	Sabzevar	0.790
**27**	Hormozgan	0.572	Neyshabur	0.550	Semnan	0.753	Shahrud	0.787
**28**	Shahid-Beheshti	0.525	Lorestan	0.531	Babol	0.750	Iran	0.783
**29**	Iran	0.524	Kurdistan	0.523	Fasa	0.748	Birjand	0.776
**30**	Neyshabur	0.519	Azerbaijan-Eeast	0.507	Jiroft	0.688	Jiroft	0.775
**31**	Qazvin	0.516	Jahrom	0.491	Ahvaz	0.685	Qazvin	0.760
**32**	Kohgiluyeh & BoyerA	0.504	Semnan	0.469	Iran	0.650	Fasa	0.753
**33**	Semnan	0.502	Iran	0.437	Birjand	0.598	ChaharM & Bakhtiari	0.748
**34**	Qom	0.499	Ahvaz	0.431	Bushehr	0.590	Jahrom	0.772
**35**	Tehran	0.480	Shahid-Beheshti	0.420	Kohgiluyeh & BoyerA	0.576	Rafsanjan	0.702
**36**	Ahvaz	0.449	Zabol	0.399	Neyshabur	0.557	Kerman	0.692
**37**	Zabol	0.406	Kashan	0.394	Gonabad	0.480	Shiraz	0.691
**38**	Kashan	0.327	Tehran	0.367	Dezful	0.435	Babol	0.673
**39**	Jiroft	0.269	Dezful	0.280	Rafsanjan	0.430	Markazi	0.663
**40**	Kerman	0.250	Kerman	0.180	Hormozgan	0.427	Semnan	0.581
**41**	Dezful	0.245	Markazi	0.155	Jahrom	0.423	Isfahan	0.573
**42**	Markazi	0.239	Yazd	0.132	Kerman	0.410	Azerbaijan-Eeast	0.546
**43**	Zahedan	0.127	Jiroft	0.082	Zabol	0.400	Ahvaz	0.525
**44**	Yazd	0.033	Zahedan	0.013	Torbat-Heidariye	0.351	Yazd	0.444
**45**	Rafsanjan	0.000	Rafsanjan	0.000	Zahedan	0.346	Kashan	0.371
**46**	Average	0.563	Average	0.559	Average	0.736	Average	0838

Table 2 represents the rank of universities in the four models used in the study and the range of rankings of each university in different models. To achieve a comprehensive agreement on the status of the performance of the universities and their position in ranking relative to each other, we used the rank of a set of ranking methods that is average of the rank of each university in different models. Gilan University with an average rank of 4.75 and Rafsanjan University with an average rank of 0.41, respectively, have obtained the first and the last rank in the performance of their health deputies. Based on the last two columns of this table in all four models, the Universities of Gilan, Ardebil, and Bojnourd, among 45 universities of the country, were ranked as the best universities, and the Universities of Rafsanjan, Kerman, Ahvaz, and Jiroft were ranked with the weakest performance.

**Table 2:**
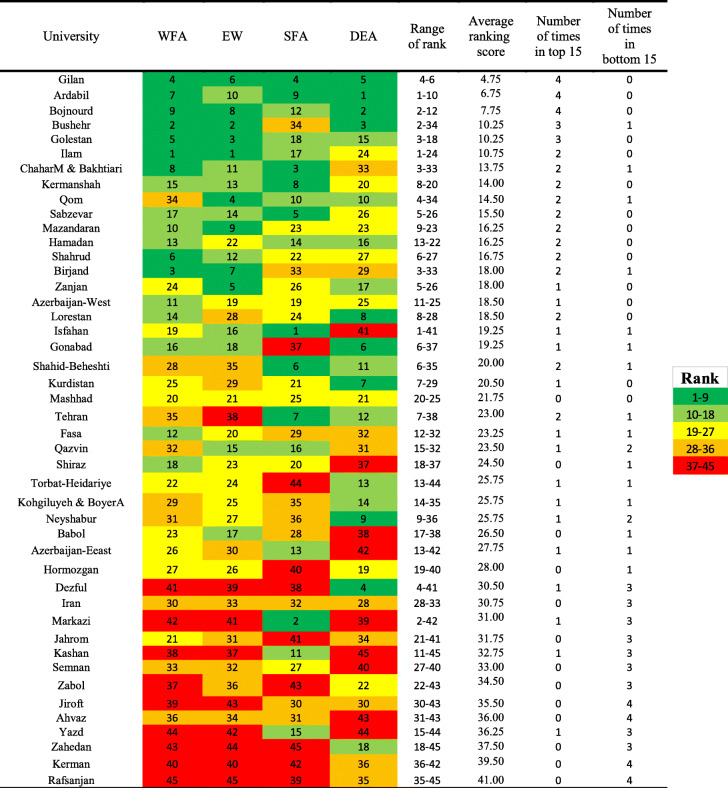
Range of rankings of universities in different model

As observed, the WFA, with a relatively high peak, is the main factor among the four models of dispersion of score performance of the universities. The WFA and EW together, and the SFA and DEA combined, reveal similar results due to their similar methodologies. According to the results obtained, the score changes are few in some universities in reference to the Universities of Ahvaz, Fasa, Gilan, Golestan, Shiraz, Shahroud, Shahid Beheshti, and Semnan, but the score changes are high in some universities such as Dezful, Jiroft, Hamedan, Rafsanjan, Torbat-e Heydariye, Yazd, Zabol, and Zahedan. The high score changes in some universities indicates the necessity for using the different models for reducing the uncertainty in their ranking and is a form of creation of reliability distance in the results of performance.

**Fig. 2:**
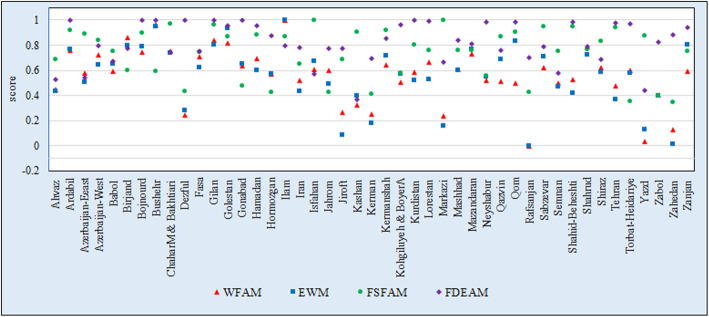
Changes of performance score in four models

Figure 2 represents the amount of changes of performance score in four models. As observed, the WFA, with a relatively high peak, is the main factor among the four models of dispersion of score performance of the universities. The WFA and EW together, and the SFA and DEA combined, reveal similar results due to their similar methodologies. According to the results obtained, the score changes are few in some universities in reference to the Universities of Ahvaz, Fasa, Gilan, Golestan, Shiraz, Shahroud, Shahid Beheshti, and Semnan, but the score changes are high in some universities such as Dezful, Jiroft, Hamedan, Rafsanjan, Torbat-e Heydariye, Yazd, Zabol, and Zahedan. The high score changes in some universities indicates the necessity for using the different models for reducing the uncertainty in their ranking and is a form of creation of reliability distance in the results of performance.

**Fig. 3:**
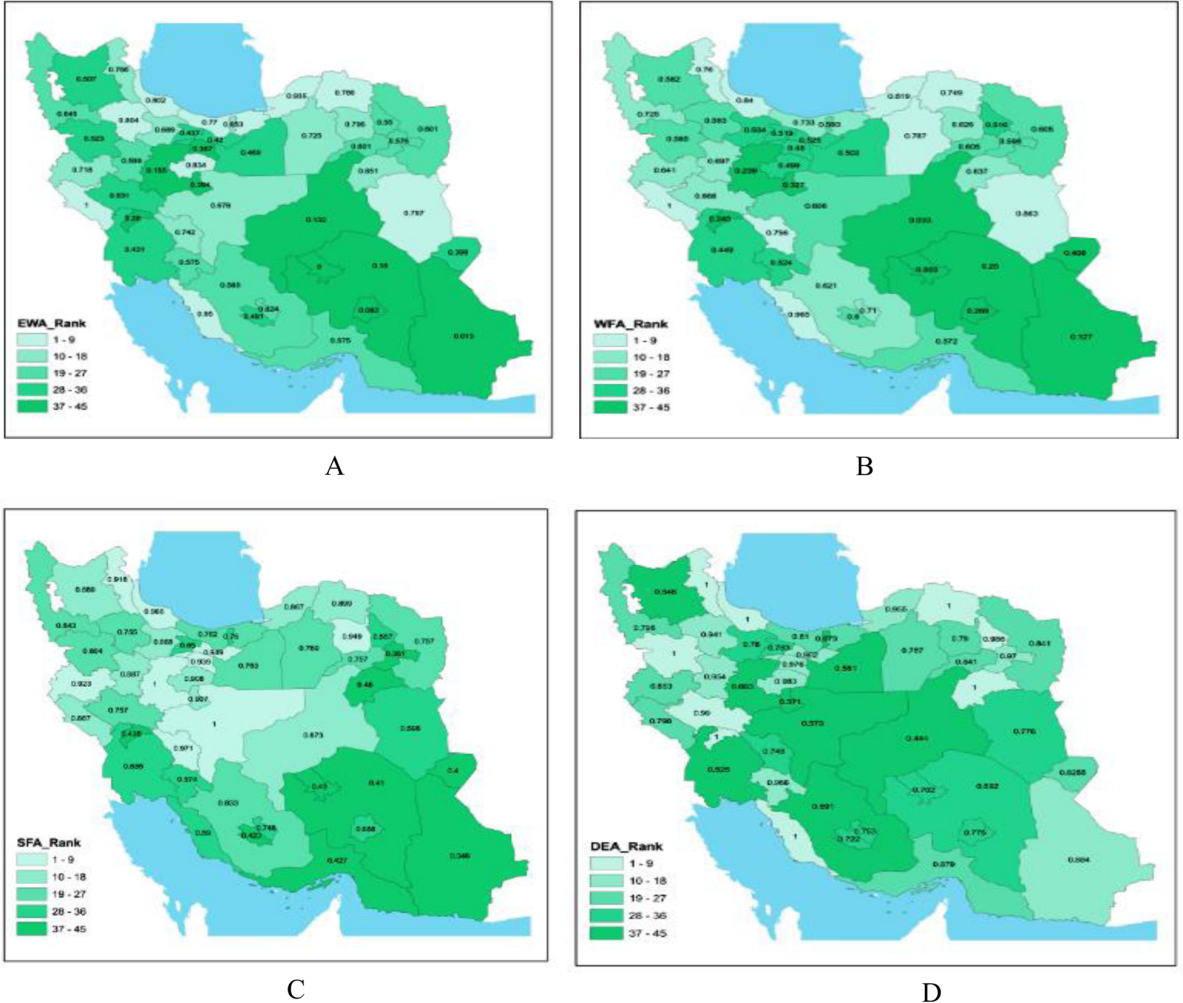
Country map of ranking the performance of universities of medical sciences in different models: A. EWM, B. WFAM, C. FDEAM, D. FSFAM. *Source: These maps are the result of this study and were drawn by the authors and not extracted from another source*

Figure 3 presents the country map of ranking the health deputies where the rank of the universities has been placed in five nine-pack packages in a green-colored spectrum; in such a way that the more bright the green color, the better the rank and status of the universities, and the darker the green color, the worse the rank and status of the universities. As observed in all four models, the universities with Smaller area represent better performance status and have better rank in comparison to the ones with larger area.

## Discussion

The present study aimed to evaluate and rank the performance of health deputies of medical sciences universities in Iran. The use of performance information for decision-making, efficiency program improvements and responsibility requires beneficiary assurance from high quality of the evaluation process and validity of its results [59, 60]. In this study, different models have been applied for the performance evaluation with the purpose of increasing result reliability.

The mean score of performance in WFA, EW, SFA and DEA was 0.563, 0.559, 0.736, and 0.838, respectively. These results indicate that individual deputies for public health of the universities of medical sciences in Iran are the main responsible persons for the PHC system and public health, who are considerably distant from access to the high level of performance score 1 in all four models, so they should take steps toward the improvement of performance by clarifying integration strategies. Considerable differences were observed in performance of health deputies at the present level of the country. The accomplished ranking Universities of Gilan, Ardebil, Bojnourd, Bushehr, and Golestan, are in the group of universities with highest performance; while the Universities of Yazd, Zahedan, Kerman, and Rafsanjan had the lowest level of performance. From among the top large universities in the country, Shiraz, Mashhad, Ahvaz, and Kerman, in the group of 15 higher universities, did not place in one of the models of the study; however, the first top large university that is observed in the ranking is Isfahan University, which secured 19th place. These results reveal that the large universities in the country, despite considerable utilization of the physical, financial, and human resources, in comparison to the other universities of the country, have no acceptable performance and should plan integrated strategies to improve the health outcomes of the population under their coverage and make efficient use of the resources in their agenda.

The universities, with low areas of scope under their coverage, have better performance status and rank in comparison to the universities with a vast area. For instance, the Universities of Kerman, Zahedan, Yazd, and Ahvaz cover a vast geographical territory and were placed in the group of 5 universities with the weakest performance. This condition is not that improbable because the provision of services in the vast areas, due to low population densities, can increase the final cost of the service provision and complicate the control and supervision of the management, as well as cause the reduction of performance level. Of course, the good performance of the universities with lesser geographical area is not general, in such a way that Rafsanjan University, despite less geographical area, has been the weakest university regarding the performance of its health deputy. The universities with greater geographical area that had weak performance have often been placed in the areas south and southeast of the country. Movahedi et al. have revealed that the suitability of indicators in the north and central provinces in Iran and their unsuitability in the eastern and south borders of the country is observed in most of the heath indicators [61]. Furthermore, according to the study of Yazdi and Mahjoub in 2011, Tehran, Gilan and Mazandaran Provinces enjoyed suitable conditions concerning the indicators of the maternal health; but Kohgiluyeh-and- Boyer-ahmad and Hormozgan Provinces did not have suitable conditions in this regard and Sistan-and-Baluchestan province had difficult conditions.

The WFA and EW represent the performance of health deputies based on their achievements without considering the production resources and. According to their results, only 65% and 55% of the universities, respectively, had obtained a score higher than the mean. Therefore, the performance of health deputies of the universities in access to the health outcomes is evaluated weak. The study of Shahraz and his colleagues indicates that Iran, in the case of the death index, which is one of the important impact indicators in the conceptual framework of the present research, has secured 12th place among 20 countries of this region [62]. It should be considered that there exists considerable difference in performance in the universities; hence, the performance score in both mentioned models varied from 1 in Ilam University to 0 in Rafsanjan University. Based on these models, the Universities of Ilam, Bushehr, Gilan, Golestan and Birjand had the best performance and Zahedan and Rafsanjan had the weakest performance. The difference in the health outcomes in different regions of the country is not limited to Iran; and such significant differences are observed in different states of the United States, as well [63, 64]. Japan also, despite the success in the reduction of mortality and debility of its citizens from 1990 to 2015, has experienced increasing differences in the health outcomes in different parts of the country [65].

The SFA and DEA have studied the achievements related to the resources and therefore, they have surveyed the performance evaluation of heath deputies in terms of efficiency. Considerable differences are observed at the country level in these models; thus, performance scores in the SFA include a range between 1 in the Universities of Isfahan and Markazi to 0.346 in Zahedan University. In the DEA model, the performance score varies from 1 in the Universities of Ardebil, Bojnourd, Bushehr, Dezful, Gilan, Gonabad, and Kordestan to 0.371 in Kashan University. A considerable part of the studies took place inside the country in the case of efficiency of health systems where focus was made on the hospitals [5, 7, 66–72] and this has indicated different degrees of inefficiency in the sector of hospital services of the country. Of the studies achieved on a comprehensive scale in regard to the evaluation of performance of the health system in Iran in the case of efficiency of all the scopes (education, research, health, and treatment), reference can be made to the study by Rashdian and his colleagues. According to those results, the average technical efficiency of the medical universities in Iran has been obtained at 0.812 using the DEA technique, which is similar to the score of 0.838 obtained in DEA of the present study [43]. Furthermore, the other studies corresponding to the present research are indicative of the existence of inefficiency in the health system of Iran. Tandon et al., in addition to the measurement of performance of health systems in 2000, have ranked Iran by a score of 0.659 in 93rd place among the countries of the world [73]. In the study completed by WHO in 2000, the health system of Iran, with a performance score of 0.805, was ranked in 58th place among 191 countries [8]. According to a study by Kumbhakar in 2010, the rank Iran has is based on a different evaluation method and has varied from 55 to 78 among 191 countries [10].

The existence of inefficiency in the health system is not limited to Iran. Around the world, there are comprehensive inefficiencies in the process of conversion of resources of health systems into the results that are the reasons for economic waste and increases in expenditures in the health systems of those countries [13]. The past studies have indicated a considerable inefficiency in developing countries and their economies [17, 74–76]. In the evaluation of performance of health systems in 173 countries of the world, the average technical efficiency of the health systems was obtained at 0.789 [77]. Furthermore, the report of WHO in 2010 has estimated that presently 20 to 40% of all health expenditures is wasted due to lack of efficiency [78].

The importance of the present research, based on the use of the two approaches of achievements and efficiency, is revealed when the results obtained from different models of the study are surveyed in a parallel form. Although Ilam University has had the best performance in the scope of achievements; when it is studied by considering the inputs in terms of efficiency, it shows a considerable reduction in performance in such a way that it has been placed 17th and 24th in rank by SFA and DEA, respectively. Although Isfahan University has not ranked as one of the 15 high-level universities in WFA and EW, it has placed 1st in SFA. The difference between the two approaches can be a suitable guide concerning planning for the improvement of performance at different universities. Universities that have performed well in models 1 and 2 (different levels of result chain including output, outcome, and impact) but have performed poorly in models 3 and 4 (efficiency) should focus on input management and efficiency improvement. On the other hand, universities that have high performance scores but have shown poor performance in Models 1 and 2 should strive to maintain optimal use of their resources. But at the same time, they need to move through benchmarking and use the experience of universities that have good levels of health indicators to improve their achievements. Universities have shown different performance in different models, from this it can be concluded that ranking based on just one model can lead to disproportionate interpretation and misunderstanding of the situation of universities in comparison with each other. In some other studies, a multi-model approach has been used, for example Mcmillan and Chan have also used this method in the efficiency rankings of Canadian universities [79].

Based on the results of the ranking used in the set of ranking methods, Gilan University has had the best performance, and Rafsanjan University has had the weakest performance among the universities of medical sciences in Iran. Although the primary health care system in Iran has been the main source of rapid and significant improvement of health indicators in recent decades [80], but this system suffers from some problems such as: mechanical organizational structure at local levels, structural mismatch with changing requirements, weak information systems. Lack of strategic management and centralized systems, lack of allocation by authorities to the local levels, weakness in comprehensiveness applications and continuation of care, lack of flexibility and responsibility, insufficient provision and inequitable distribution of resources, and lack of motivation in the payment system [81–83] can also be reasons, in the present study, for weak performance of the health deputies. Most of the problems of the health system in Iran, such as the health deputies, result from the lack of integrated systems of information management [84] and the lack of a system of monitoring and performance evaluation. Although many particular efforts have been made in the last years for evaluating the performance of health deputies, this matter has not been perpetuated and it has many shortcomings (46). The evaluation and assessment of the programs is neither perfect nor systematic, so, many efforts should be made to create a comprehensive informational integrated system [35]. The evaluation of existing programs and creation of health-centered competition between the provinces and cities are measures needed to access better outcomes and performance in the case of health deputies [85]. The results of the present study, and the models used, can be a basis for beginning the periodical evaluations in the structure of system of primary health care in the country.

Each of the used models has the required accuracy in the method and calculations, as well as validity in the results. Each model has a separate and valuable message in terms of its unique assumptions and features. Nevertheless, considering the following reasons, Model 3 is a more appropriate option for evaluating the performance: this model looks at the performance of universities as a production function that seeks to influence the outcomes of the activities and services of universities. The number of initial indicators used in Model 3 is more than others and includes 53 indicators. This number of indicators shows that more variables have been included in the performance measurement and therefore the results seem more realistic because of more dimensions and activities of public health deputies. Socio-economic indicators (Urbanization, Income of urban and rural households, Years of education, Geographical area covered) as random and uncontrollable components by universities but affecting their performance, along with the inefficiency component of them form the structure of Model 3. Therefore, it seems this model in comparison with others has more comprehensiveness and logic.

This study is the first comprehensive PHC and public health systems ranking study in Iran. Strong points of this study can be mentioned regarding the use of a domestic conceptual framework that has been designed based on the structure of the system of public and primary health care, and its specific programs in the country using four models in the evaluation of the health deputies. The assumptions and different structures of these models from different viewpoints resulted in the study of performance. The calculated composite index has provided the possibility of reflection on a wide set of indicators at different levels of results-chains in the final findings of this study. In fact, the method used in the study has reduced the amount of uncertainty and increased the reliability of the study results by applying an analysis of wide sensitivity. This study had some limitations, however, such as the problem of incomplete or unrecorded data and being regional based instead of university in part of the existing data. Although in most of the provinces of the country, one medical sciences university is responsible for the presentation of health services to all the population of that province; some provinces such as Tehran, Fars, North Khorasan, Kerman and others having several medical sciences universities. Therefore, access to some data separately sorted by the medical sciences universities in these provinces was not possible. According to the wide social-economic, geographical, and cultural differences existing in the country, the self-comparison performance of the universities could give a better viewpoint about the performance of universities to the policymakers. Due to the data restriction, this study was only based on the data of one year. In this study, some dimensions of performance such as social participation and population satisfaction, cross-sectoral coordination, employee satisfaction and commitment, educational processes and financial resource management are not considered. The approach of the present study has been to use the results chain framework to measure performance, and our assumption has been that all of the above dimensions are ultimately reflected in this framework. Therefore, the above limitations do not affect the acceptability of the results of this study.

The perception of complex epidemics and the determination of efficiency and effectiveness of the programs in the domain of public health require a continuous, comprehensive, strategic and multi-method evaluation and monitoring system. The national programs of monitoring and evaluation should be regularly studied and updated so that they could be in accordance with the changes in the national strategic programs, and the performance of public health systems could be improved based on the results of periodical evaluations of the evaluation and monitoring system [86]. Ranking the sub national health systems, as was accomplished in the present study, improves the policies related to the clarity and collection of the data and consequently. The results of such ranking can provide a valuable opportunity for the improvement and considerable motivation for the stimulation of planning processes, as well as provide the possibility of learning from regions having better performance [21].

## Conclusions

The average performance of primary health care and public health in universities of medical sciences at the country level does not possess suitable conditions. Of course, these conditions do not relate to all the universities and there is much dispersion in the performance of universities at this level. The top large universities of the country have had weak performance despite having considerable resources. Therefore, managers of the universities must improve their accountability regarding the methods they use to govern resources, and the amount of achievement that can be seen in their results. The universities with greater geographical area, but limited level of performance, should regulate the method of organizing their services in such a way that it facilitates the control of system performance and the improvement in the level of health indicators in all areas under their jurisdiction. With regard to variation between the levels of performance of universities in different models, every university should apply a special strategy that is suitable for its specific condition of performance. It is suggested that the methodology of the present study be used for annual ranking of the health deputies in the country.

## Data Availability

The datasets supporting the conclusions of this article are included within the article/tables. The raw data can be requested from the corresponding author on reasonable request.

## Abbreviations

WHO: World health organization
WFA: Weighted factor analysis model
EW: Equal weighting model
SFA: Stochastic frontier analysis
DEA: Factor and data envelopment analysis
MOHME: Ministry of health and medical education
SCI: Statistical center of Iran
PHC: Primary health care
